# Time after time: atherosclerotic coronary artery disease after a normal CT angiogram in SCOT-HEART

**DOI:** 10.1007/s00330-026-12454-2

**Published:** 2026-03-05

**Authors:** Anne-Marieke Stantien, Ann-Christine Stahl

**Affiliations:** Department of Radiology, Charité - Universitätsmedizin Berlin, Corporate Member of Freie Universität Berlin and Humboldt-Universität zu Berlin, Berlin, Germany

Coronary CT angiography (CTA) has become a cornerstone of contemporary cardiovascular imaging for the identification and quantification of atherosclerotic coronary artery disease (CAD) [1] and is now firmly embedded in European and American guidelines [2, 3]. Large, randomized trials, including SCOT-HEART and DISCHARGE, have demonstrated that CTA improves diagnostic certainty, reduces unnecessary invasive coronary angiography (ICA), lowers procedural risks of subsequent management, and enables earlier initiation of preventive therapies in patients with stable chest pain [4, 5]. As CTA has moved from innovation to implementation, an inevitable next question has emerged: what happens over time to patients whose coronary arteries are initially normal, showing no signs of atherosclerotic CAD? There are large observational studies in asymptomatic individuals, including serial imaging, and data suggest a time span of at least 5 years for repeat scanning upon a calcium score of 0 [6]. However, no randomized trial has previously reported CAD findings a decade after an initial normal CTA.

Routine serial coronary imaging is neither recommended nor desirable in routine clinical practice, given concerns about radiation exposure and the risk of triggering unnecessary downstream testing. However, understanding the temporal trajectories of CAD remains clinically and biologically important, as progression has traditionally been inferred only indirectly from symptoms and clinical events. Therefore, the analysis presented as a late-breaking clinical trial in radiology at the European Congress of Radiology and published simultaneously today in the *European Radiology* journal, leverages a unique opportunity within the SCOT-HEART trial: patients with no signs of atherosclerotic CAD at baseline who underwent clinically indicated CT imaging during long-term follow-up [7]. These data provide rare randomized-trial-anchored insights into the emergence of coronary atherosclerosis over a 10-year period and into the clinical consequences of detecting disease once a diagnosis of “normal” has already been made.

To appreciate the implications of this analysis, it is essential to consider how follow-up imaging occurred. Among the 524 patients who had no signs of CAD at baseline, repeat CTA was performed in a small subset of patients (*n* = 31 (6%); *n* = 30 with contrast), whereas a larger number received non-gated chest CT (*n* = 162 (31%)) for a variety of clinical indications. Patients who underwent chest CT compared to CTA had lower rates of detected CAD, with just one patient having severe calcifications (*n* = 1/162 (0.6%)), while most presented with moderate (*n* = 30/162 (24%)) or no calcifications (*n* = 121/162 (75%)). By contrast, almost half of patients undergoing repeat coronary CTA developed atherosclerotic CAD, although only a small subgroup developed obstructive CAD ≥ 50% stenosis (*n* = 4/30 (13%)); 36% had non-obstructive CAD (*n* = 11/30), and 53% showed no CAD (*n* = 16/30). Repeat CTA was prompted by recurrent or persistent symptoms, enriching this group for clinically manifest disease, whereas chest CT provided a more incidental, less symptom-driven assessment of the coronary arteries. At the same time, the comparison of CTA and chest CT may also influence this result, as CTA allows the visualization of different plaque types and chest CT only of calcified lesions. When both imaging modalities are considered together, approximately one-third of patients with initially normal coronary arteries were found to have developed signs of CAD over a median follow-up exceeding eight years. The overall added patient number with newly developed obstructive CAD or severe calcifications, however, remains low with *n* = 5, and most patients in whom atherosclerotic CAD developed presented with mild- to moderate disease severity.

Notably, the detection of CAD on subsequent CT was not associated with an increase in all-cause mortality, but it was associated with a substantially higher likelihood of ICA. This difference between clinical outcomes and invasive procedures reflects a broader challenge in contemporary health care. Rather than prompting timely initiation or intensification of preventive therapy, the detection of CAD too often accelerates patients on their path toward the catheter laboratory. The potential overuse of diagnostic testing is a cause for concern. Redundant coronary imaging, such as a CTA sometimes followed by diagnostic ICA irrespective of findings, adds cost, exposes patients to harm, and risks conflating anatomical disease with an indication for intervention. But then, why do patients with documented disease so frequently receive invasive procedures? Symptoms undoubtedly play a role, but symptom relief should not be confused with prognostic benefit. A patient with no symptoms and a high plaque burden may profit more from a statin medication than a PCI. But when should preventive therapy be initiated? Perhaps CTA-derived individualized treatment plans, based on percentile curves of plaque volume, could be more effective than current risk-based strategies [8]. New treatment algorithms, for example, the treatment of any coronary plaque with lipid-lowering medication as recommended by the Quantitative Cardiovascular Imaging study group, merit prospective evaluation [8]. If one-third of plaque-free patients develop atherosclerotic CAD within a decade, waiting for symptoms may miss the sweet spot while risk factors promote plaque progression [9]. The task ahead is therefore not to image more, but to identify when and whom to image and which patients should enter preventive pathways without the need for repeated imaging.

Serial imaging derived from large, randomized trials offers a rare glimpse into patients’ trajectories within the health care system and can inform improvements in risk stratification, guideline development, and health-economic decision-making. It also highlights the importance of structured long-term follow-up in randomized CTA trials. As this study represents an essential first step showing us how long-term CAD trajectories can be studied, it is an excellent basis for the long-term follow-up planned in SCOT-HEART EVOLUTION and DISCHARGE-Extend. Atherosclerotic CAD is a condition of time, and CTA is not an endpoint but a snapshot of disease burden along a dynamic continuum. As CTA continues to be implemented more widely in clinical practice, the challenge lies not in imaging itself, but in what management follows imaging. We must better define when preventative therapies should be initiated—early enough to alter trajectories yet targeted enough to avoid overtreatment—when follow-up imaging is justified, and how to prevent the overuse of both noninvasive and invasive diagnostic strategies. Ultimately, if CTA is to fulfill its promise, precision in diagnosis must be matched by precision in prevention and treatment, time after time.

## References

[1] Zaman S, Wasfy JH, Kapil V et al (2025) The *Lancet* Commission on rethinking coronary artery disease: moving from ischaemia to atheroma. Lancet 405:1264–1312. 10.1016/S0140-6736(25)00055-8 40179933 10.1016/S0140-6736(25)00055-8 PMC12315672

[2] Vrints C, Andreotti F, Koskinas KC et al (2024) 2024 ESC guidelines for the management of chronic coronary syndromes: developed by the task force for the management of chronic coronary syndromes of the European Society of Cardiology (ESC) and endorsed by the European Association for Cardio-Thoracic Surgery (EACTS). Eur Heart J 45:3415–3537 39210710 10.1093/eurheartj/ehae177

[3] Virani SS, Newby LK, Arnold SV et al (2023) 2023 AHA/ACC/ACCP/ASPC/NLA/PCNA guideline for the management of patients with chronic coronary disease: a report of the American Heart Association/American College of Cardiology Joint Committee on clinical practice guidelines. Circulation 148:e9–e119 37471501 10.1161/CIR.0000000000001168

[4] SCOT-HEART Investigators (2018) Coronary CT angiography and 5-year risk of myocardial infarction. N Engl J Med 379:924–933 30145934 10.1056/NEJMoa1805971

[5] DISCHARGE Trial Group (2022) CT or invasive coronary angiography in stable chest pain. N Engl J Med 386:1591–1602 35240010 10.1056/NEJMoa2200963

[6] Gopal A, Nasir K, Liu ST et al (2007) Coronary calcium progression rates with a zero initial score by electron beam tomography. Int J Cardiol 117:227–231. 10.1016/j.ijcard.2006.04.081 16875746 10.1016/j.ijcard.2006.04.081

[7] Avigdor L, Williams S, Guimaraes AR et al (2026) Development of coronary artery disease in patients with initially normal coronary arteries in the SCOT-HEART trial. Eur Radiol. 10.1007/s00330-026-12353-6 10.1007/s00330-026-12353-6 PMC13282284 41781727

[8] Schulze K, Stantien A, Williams MC et al (2026) Coronary CT angiography evaluation with artificial intelligence for individualized medical treatment of atherosclerosis: a consensus statement from the QCI Study Group. Nat Rev Cardiol 23:100–115 40751112 10.1038/s41569-025-01191-6

[9] Lan Z, Ding X, Yang W et al (2025) Impact of lipoprotein (a) on coronary atherosclerosis and plaque progression in patients with type 2 diabetes mellitus. Eur Radiol 35:1533–1542. 10.1007/s00330-024-11313-2 39945810 10.1007/s00330-024-11313-2

